# Inter-platform concordance of gene expression data for the prediction of chemical mode of action

**DOI:** 10.1186/s13062-016-0167-9

**Published:** 2016-12-20

**Authors:** Chathura Siriwardhana, Susmita Datta, Somnath Datta

**Affiliations:** 1 Office of Biostatistics & Quantitative Health Sciences, University of Hawaii John A. Burns School of Medicine, Honolulu, 96813 HI USA; 2 Department of Biostatistics, University of Florida, Gainesville, 32603 FL USA

**Keywords:** Classification, Microarray, RNASeq

## Abstract

**Background:**

It is interesting to study the consistency of outcomes arising from two genomic platforms: Microarray and RNAseq, which are established on fundamentally different technologies. This topic has been frequently discussed from the prospect of comparing differentially expressed genes (DEGs). In this study, we explore the inter-platform concordance between microarray and RNASeq in their ability to classify samples based on genomic information. We use a set of 7 standard multi-class classifiers and an adaptive ensemble classifier developed around them to predict Chemical Modes of Actions (MOA) of data profiled by microarray and RNASeq platforms from Rat Liver samples exposed to a variety of chemical compounds. We study the concordance between microarray and RNASeq data in various forms, based on classifier’s performance between two platforms.

**Results:**

Using an ensemble classifier we observe improved prediction performance compared to a set of standard classifiers. We discover a clear concordance between each individual classifier’s performances in two genomic platforms. Additionally, we identify a set of important genes those specifies MOAs, by focusing on their impact on the classification and later we find that some of these top genes have direct associations with the presence of toxic compounds in the liver.

**Conclusion:**

Overall there appears to be fair amount of concordance between the two platforms as far as classification is concerned. We observe widely different classification performances among individual classifiers, which reflect the unreliability of restricting to a single classifier in the case of high dimensional classification problems.

**Reviewers:**

An extended abstract of this research paper was selected for the Camda Satellite Meeting to Ismb 2015 by the Camda Programme Committee. The full research paper then underwent two rounds of Open Peer Review under a responsible Camda Programme Committee member, Lan Hu, PhD (Bio-Rad Laboratories, Digital Biology Center-Cambridge). Open Peer Review was provided by Yiyi Liu and Partha Dey. The Reviewer Comments section shows the full reviews and author responses.

## Background

For more than a decade microarray technology has provided enormous momentum to the modern genomic research. The ability of quantify thousands of genes’ expressions at the same time has led to remarkable achievements in wide range biological studies. Abundance of microarray assays has been published worldwide in various databases. However, microarray technology has some limitations, such as the accuracy of expression measurements limited by levels of hybridization and variability hybridization properties of probes [1]. RNAseq is a version of next generation sequencing technology which has recently become popular due to some of its advancement over the microarray technology. Evidently, RNASeq has a potential advantage in measuring absolute expression levels compared to the microarray technique [2, 3]. Since these two methods fundamentally differ in their underline technologies, it is interesting know if this disparity results an inconstancy in experimental outcomes. Classifiers are known to be one of the most widely use statistical tools in genomic oriented biomedical studies. For an example, identifying at risk individuals for a certain disease type such as cancers, based on their genetic profiles. In this work, we explore the concordance between microarray and RNASeq genomic platforms in the context of classifications based on a set of comparative classification experiments carried using these two platforms.

In recent years, a number of authors have discussed the agreement between scientific conclusions made on microarray and RNASeq platforms, based on comparative analyses. A common choice for these studies was the concordance of differentially expressed genes (DEGs). A previous study that described a large scale comparison of microarray and RNASeq platforms using the Cancer Genome Atlas (TCGA) based analysis, reported a high correlation among expressions levels resulted from both platforms and suggested a reasonable concordance between DEGs by comparing tumors with normal tissues [4]. Another study compared these two bases using an analysis on data obtained from a colon cancer study and conclude that RNASeq had an advantage over microarray for detecting DEGs [5]. A recent article provided a comprehensive assessment between microarray and RNASeq methods, comparing DEGs using gene expressions resulted from a rat liver experiment [6]. Further they described the concordance in aspect of classification assessing the predictability of classes defined by the chemical mode of action (MOA), using a set of classifiers trained in two genomic platforms. Their study revealed weak classification accuracies for a set of classifiers when applied to these platforms.

Our work is based on the previously described rat liver data [6], where we primarily focus on developing a common classifier that works reasonably well in cross platforms providing better predictability. Next, we discuss the concordance between microarray and RNASeq platforms in various forms in prospect of classification. Furthermore, we identify a set of important genes for specifying classes given by MOAs by focusing their effects on the classifier accuracy. We use seven standard classifiers and an adaptive ensemble classifier built around them to achieve these goals. This study is part of the 2015 annual conference on Critical Assessment of Massive Data Analysis (CAMDA) challenges. The Rat liver experiment was conducted by the FDA SEQC consortium to assess the performance of modern gene transcript expression profiling methods, which is a comparative analysis designed for developing predictive models to predict the chemical mode of action (MOA).

The remainder of the article is organized as follows. In Section “Results”, we provide results and conclusions of the study. Section “Methods” explains all underline procedures applied. The main body of the paper ends with a discussion in Section “Discussion”.

## Results

### Classification in individual platforms

**Fig. 1:**
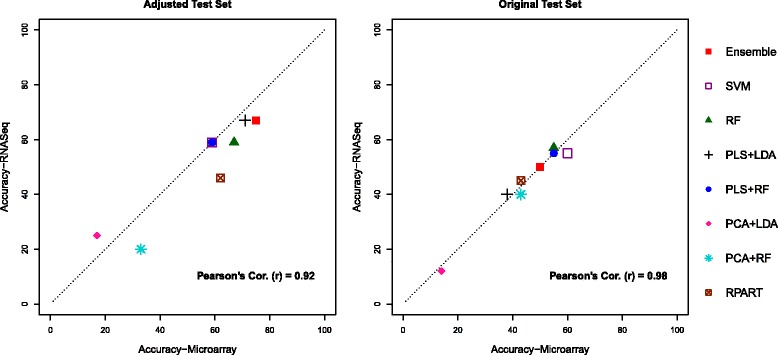
Plots between prediction accuracies of RNASeq vs Microarray for two different test sets using the common gene set, by eight different classification techniques, for classifiers trained and predicted on individual platform

**Fig. 2:**
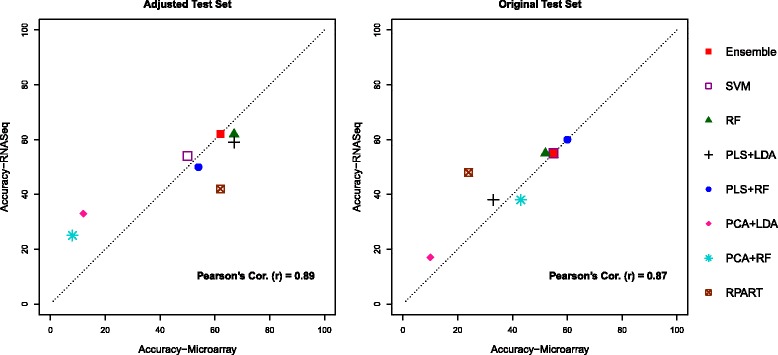
Plots between prediction accuracies of RNASeq vs Microarray for two different test sets using the complete gene set, by eight different classification techniques, for classifiers trained and predicted on individual platform

We first describe outcomes of the Analysis 1, which was performed using two basic strategies: adjusted and originally given test sets described in Section “Methods”. We provide a detailed summary of these results in Tables 1, 2, 3 and 4, where each table presents the classifier’s overall prediction accuracy, class specific sensitivity and the corresponding specificity. Graphical representations of the summarized result are also provided on Figs. 1 and 2.

**Table 1:** Accuracies of predicting MOA’s in the adjusted test set, based on classifiers developed on gene expression sets profiled from microarray and RNASeq platforms

Platform	Classifier	Overall Acc. %	Sensitivity, Specificity		
			PPARA	CAR/PXR	Control
Microarray	Ensemble	75	89,67	44,94	100,67
	SVM	58	56,59	33,73	100,44
	RF	67	89,54	22,94	100,56
	PLS+LDA	71	67,73	56,80	100,61
	PLS+RF	58	44,68	44,68	100,45
	PCA+LDA	17	0,27	0,27	67,0
	PCA+RF	33	33,33	0,53	83,16
	RPART	62	100,39	11,93	83,55
RNASeq	Ensemble	67	56,74	56,74	100,56
	SVM	58	67,54	22,81	100,45
	RF	58	67,54	22,81	100,45
	PLS+LDA	67	56,74	56,74	100,56
	PLS+RF	58	67,54	22,81	100,45
	PCA+LDA	25	33,20	0,40	50,17
	PCA+RF	20	22,19	11,25	33,16
	RPART	46	22,60	33,54	100,28

**Table 2:** Accuracies of predicting MOA’s in the originally given test set, based on classifiers developed on common gene expression sets profiled from microarray and RNASeq platforms

Platform	Classifier	Overall Acc. %	Sensitivity, Specificity			
			PPARA	CAR/PXR	Control	OTHER
Microarray	Ensemble	50	44,52	44,52	100,42	39,58
	SVM	55	0,70	0,70	83,50	100,21
	RF	57	0,73	0,73	100,50	100,25
	PLS+LDA	40	67,33	56,36	67,36	6,67
	PLS+RF	55	11,67	11,67	100,48	83,34
	PCA+LDA	12	0,15	0,15	83,0	0,21
	PCA+RF	40	0,51	0,51	94,31	0,70
	RPART	45	100,30	0,57	83,39	28,58
RNASeq	Ensemble	50	33,55	33,55	100,42	50,50
	SVM	60	0,76	11,73	100,53	100,30
	RF	55	0,70	0,70	100,48	94,26
	PLS+LDA	38	56,33	56,33	100,28	0,66
	PLS+RF	55	11,67	22,64	100,48	78,38
	PCA+LDA	14	0,18	0,18	100,0	0,24
	PCA+RF	43	0,55	0,55	83,36	72,21
	RPART	43	33,46	33,46	33,45	56,33

**Table 3:** Accuracies of predicting MOA’s in the adjusted test set, based on classifiers developed on complete gene expression sets profiled from microarray and RNASeq platforms

Platform	Classifier	Overall Acc. %	Sensitivity, Specificity		
			PPARA	CAR/PXR	Control
Microarray	Ensemble	62	56,66	44,73	100,49
	SVM	50	33,60	44,54	83,39
	RF	67	89,54	22,94	100,56
	PLS+LDA	67	67,67	44,81	100,56
	PLS+RF	54	44,60	33,67	100,39
	PCA+LDA	12	33,0	0,20	0,17
	PCA+RF	8	22,0	0,13	0,11
	RPART	62	100,39	11,93	83,55
RNASeq	Ensemble	62	56,66	44,73	100,49
	SVM	54	44,60	33,67	100,39
	RF	62	78,52	22,86	100,49
	PLS+LDA	58	44,66	44,66	100,44
	PLS+RF	50	44,54	22,67	100,33
	PCA+LDA	33	33,33	0,53	83,16
	PCA+RF	25	22,27	0,40	67,11
	RPART	42	44,41	22,54	67,34

**Table 4:** Accuracies of predicting MOA’s in the originally given test set, based on classifiers developed on complete gene expression sets profiled from microarray and RNASeq platforms

Platform	Classifier	Overall Acc. %	Sensitivity, Specificity			
			PPARA	CAR/PXR	Control	OTHER
Microarray	Ensemble	55	33,61	44,58	100,48	56,54
	SVM	55	0,70	0,70	83,50	100,21
	RF	55	0,70	0,70	83,50	100,21
	PLS+LDA	38	67,30	44,36	100,28	0,66
	PLS+RF	60	11,73	0,76	100,53	100,30
	PCA+LDA	17	0,22	11,19	83,6	6,25
	PCA+RF	38	0,48	0,48	0,44	89,0
	RPART	48	100,34	0,61	83,42	33,59
RNASeq	Ensemble	55	44,58	33,61	100,48	56,54
	SVM	55	0,70	0,70	83,50	100,21
	RF	52	0,66	0,66	83,47	94,20
	PLS+LDA	33	44,30	44,30	100,22	0,58
	PLS+RF	60	0,76	22,70	100,53	94,34
	PCA+LDA	10	0,13	22,7	33,6	0,18
	PCA+RF	43	0,55	0,55	17,47	94,5
	RPART	24	44,19	22,25	67,17	0,42

We first discuss the classification resulted from using a set of genes that are represented in both platforms. For the adjusted test set, the left panel of the Fig. 1 shows that the performance of each classifier is similar in both platforms, since all the data points are fairly close to the diagonal line (Pearson’s *r*=0.92). The accuracy of individual classifier varies from 17 to 75%, and as to be expected, the performance of the ensemble classifier is the best in both platforms. The overall accuracy of the optimal classification method is slightly better in microarray compared to RNA-seq (75% vs 67%). In particular, we observe a lower prediction accuracy for the class “PPARA" in RNASeq (56%), compared to the microarray (89%) platform. Overall, the class given by “CAR/PXR" which has a maximum sensitivity of only 56%, seems to be the MOA that hardest to predict. Some individual classifiers show widely different prediction sensitivity for the same class in two platforms. For example the sensitivity for “PPARA" by RPART is 100% in microarray, whereas it reaches as low as 22% in RNAseq.

When the original (i.e., unadjusted) test set is used, we again observe matching performance of classifiers in both platforms (Table 2) similar to the case with the adjusted test set; in fact, the agreement is even higher (Pearson’s *r*=0.94) as shown in the right panel of the Fig. 1. The overall accuracy ranges from 60 to 12% indicating a drop in the classification performance compare to the previous scenario. For example, 75% vs 50% in microarray and 67% vs 50% in RNASeq for the ensemble classifier. Comparing Tables 1 and 2, we also notice a decline in sensitivities of predicting three known classes namely “PPARA", “CAR/PXR", and “Control". Since this analysis was carried using an alternative approach as described in the Section “Methods”, such decline could be possibly resulted from classifying several samples belonging to above known classes as “OTHER" by depressing the “true" class probability below 0.5 if these class attributes are somewhat close to one another. In this case, few other individual classifiers such as SVM, RF outperform the ensemble classifier in terms of the overall accuracy. But nevertheless, the ensemble classifier still acts as the best overall amongst all with regard to all performance measures.

Even with the complete set of genes, we observe similar conformity of classifiers’ performance between the two platforms (Fig. 2) as described above. Specifically for the ensemble classifier the overall accuracy is identical in the two platforms, in each case. According to Tables 3 and 4, the overall accuracy ranges between 8 to 67% and 10 to 55%, for adjusted test set and the original test set, respectively. Even though we used bigger gene sets, there is no additional improvement for predicting MOAs; indeed the performance gets worse, which is quite evident for the adjusted test set. However, some classifiers surprisingly hold equal performances for both sets of genes. As for example, the RPART shows identical performances in the microarray platform under bigger and smaller sets of genes.

### Classification in cross platforms

**Fig. 3:**
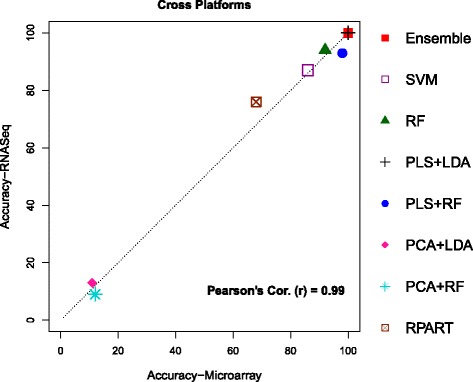
Plots between prediction accuracies of RNASeq vs Microarray test sets, by eight different classification techniques, for classifiers trained and predicted on cross platforms

Results of the 2nd analysis, namely, classification in cross platform are summarized in Table 5 and Fig. 3. We performed this study using only the common set of genes since both platforms are involved together throughout the analysis. Compared to all previous classifications we discussed in Analysis 1, this result shows even greater agreement between the prediction accuracies of the classifiers trained on a bigger training set in one platform and used to predict using the bigger test data on the other platform (Pearson’s *r*=0.99). Remarkably, the ensemble classifier was able to provide 100% accurate predictions for both cases, regardless of the additional complexity caused by 8 varieties of classes. In this analysis, the component classifier PLS+LDA also performed similarly to the ensemble classifier in both cases yielding 100% accurate class predictions. Apart from above two classifiers, SVM, RF, and PLS+RF also hold substantially high prediction accuracies.

**Table 5:** Accuracies of predicting MOA’s in the whole datasets (inducing testing and training sets) of RNAseq and microarray platforms, using the classifiers trained on corresponding opposite platform

Procedure	Classifier	Overall Acc. %	Sensitivity, Specificity							
			PPARA	CAR/PXR	AhR	Cytotoxic	DNADamage	ER	HMGCOA	Control
Trained on microarray and predicted on RNASeq	Ensemble	100	100,100	100,100	100,100	100,100	100,100	100,100	100,100	100,100
	svm	86	100,83	100,83	33,91	44,99	67,88	89,86	100,85	100,82
	RF	92	100,90	100,90	56,95	89,93	67,94	100,91	100,91	100,90
	PLS+LDA	100	100,100	100,100	100,100	100,100	100,100	100,100	100,100	100,100
	PLS+RF	98	100,98	100,98	78,100	100,97	100,98	100,98	100,98	100,97
	PCA+LDA	11	0,13	6,12	44,8	0,14	0,12	0,12	0,12	29,6
	PCA+RF	12	0,15	6,13	0,13	0,16	0,13	0,13	0,13	50,1
	RPART	68	83,65	61,69	33,71	67,68	67,68	67,68	100,65	62,70
Trained on RNASeq and predicted on microarray	Ensemble	100	100,100	100,100	100,100	100,100	100,100	100,100	100,100	100,100
	svm	87	100,84	94,86	22,93	75,88	80,88	89,87	100,86	100,83
	RF	94	100,93	100,93	78,96	88,95	87,95	78,96	100,93	100,92
	PLS+LDA	100	100,100	100,100	100,100	100,100	100,100	100,100	100,100	100,100
	PLS+RF	93	94,93	100,92	78,94	100,92	87,94	78,94	100,92	97,92
	PCA+LDA	13	0,16	0,16	0,14	0,14	0,14	0,14	0,14	47,3
	PCA+RF	9	0,11	0,11	22,8	0,10	0,10	0,10	0,10	30,3
	RPART	76	100,71	94,72	0,83	100,74	68,77	100,74	0,83	87,73

Exploring outcomes resulted from Analysis 1 and 2 (Tables 1, 2, 3, 4 and 5), we clearly notice, between the two types of dimension reduction methods, PLS performs far better than PCA throughout this study. The performances of classifiers integrated with PCA are clearly the weakest among all individual classifiers in each scenario.

### Importance of genes

We summarize results of the 3rd analysis in Tables 6, 7, 8 and 9, where each table lists the top 20 important gene name and the overall accuracy obtained by the cross validation. As we describe in the methods section this analysis was performed using two experiments: (i) using the adjusted test set and (ii) the full dataset. Furthermore, we consider using the common and complete sets of genes as additional sub-analyses within above primary experiments.

**Table 6:** Genes ranked by the importance based on accuracy reduction, for Microarray and RNA-Seq, using the adjusted test set with the common set of genes

Rank	Microarray		RNA-Seq	
	Gene name	Resulted accuracy	Gene name	Resulted accuracy
1	Cyp1a1	0.561	Fam111a	0.540
2	RT1-Bb	0.575	Evc	0.610
3	Fam111a	0.585	Cyp1a1	0.621
4	Ugt2b	0.599	Cyp1a2	0.625
5	Aldh1a7	0.628	Akr1b8	0.625
6	Akr1b8	0.632	Hbb	0.625
7	Gpnmb	0.647	Ugt2b	0.625
8	Obp3	0.647	Dhrs7	0.626
9	Hbb	0.649	Mme	0.628
10	Vnn1	0.658	Nr1d1	0.628
11	Tsku	0.660	Cish	0.631
12	Aldh1a1	0.668	Abcc3	0.631
13	RGD1309362	0.668	Adora1	0.632
14	Socs2	0.669	Fos	0.636
15	LOC685020	0.671	Abcd2	0.640
16	Aldh1b1	0.672	Irs3	0.642
17	RGD1564865	0.674	Asrgl1	0.644
18	Cyp1a2	0.675	Pilra	0.646
19	Psat1	0.676	Ddhd1	0.646
20	Gadd45g	0.678	Ugt2b17	0.647

**Table 7:** Analysis 3: Genes ranked by the importance, for microarray and RNASeq, using the adjusted test set with complete sets of genes

Rank	Microarray		RNA-Seq	
	Gene name	Resulted accuracy	Gene name	Resulted accuracy
1	Fam111a	0.572	Abcb1b	0.551
2	Abcc3	0.606	GTP_EFTU_D3.1	0.563
3	Adam8	0.624	Hba-a2	0.564
4	LOC100911107 0.628		Hbb	0.569
5	Atf3	0.632	Cyp1a1	0.569
6	Krt10	0.635	LOC360504	0.572
7	Aldh1a7	0.638	Casp12	0.572
8	MGC108823	0.638	Ugt2b	0.572
9	Ckap2	0.638	Apof	0.575
10	Cyp1a1	0.638	MGC72973	0.578
11	Asrgl1	0.639	blarkly	0.578
12	Hamp	0.640	Dhrs7	0.578
13	Hbb	0.640	Laminin_G_2.1	0.579
14	Angptl4	0.640	LOC313220	0.579
15	Oas1a	0.640	Car3	0.579
16	Psat1	0.640	Dbp	0.579
17	Igfbp2	0.642	Mcm5	0.581
18	Gsta3	0.643	TCTP.0	0.581
19	Obp3	0.649	Egln3	0.581
20	Pik3r1	0.649	Fam111a	0.581

**Table 8:** Genes ranked by the importance (based on the measure given by *R*), for Microarray and RNA-Seq, using the whole data including 8 verities of MOAs with the common gene set

Rank	Microarray			RNASeq		
	Gene name	Resulted accuracy	*R*	Gene name	Resulted accuracy	*R*
1	Cyp1a1	0.9538	0.0064	Cyp1a1	0.9658	0.0063
2	RT1-Bb	0.9707	0.0018	Abcc3	0.9786	0.0019
3	Gstp1	0.9740	0.0017	Cyp7a1	0.9689	0.0016
4	Usp2	0.9600	0.0015	Cyp1a2	0.9751	0.0016
5	Nr1d1	0.9693	0.0012	Fabp2	0.9705	0.0015
6	Obp3	0.9694	0.0011	Sgcb	0.9677	0.0014
7	Fam111a	0.9733	0.0011	Atf3	0.9672	0.0014
8	Prss23	0.963	0.0009	Gdf15	0.9692	0.0013
9	Igtp	0.9668	0.0009	Apoa4	0.9699	0.0011
10	Taf8	0.9725	0.0008	Slc13a3	0.9751	0.0011
11	Dmbt1	0.9768	0.0008	Ugt2b17	0.9751	0.0011
12	Ccng1	0.9611	0.0008	Acy3	0.9670	0.0011
13	Cav1	0.9654	0.0008	Porcn	0.9732	0.0011
14	Rnf152	0.9697	0.0008	Slc7a5	0.9652	0.0011
15	Cxcl10	0.9711	0.0008	Hdc	0.9676	0.0010
16	Rhbdf2	0.9764	0.0008	Ddhd1	0.9686	0.0010
17	Casp4	0.9683	0.0008	Rprm	0.9743	0.0010
18	Cyp2c12	0.9688	0.0008	Btg3	0.9700	0.0010
19	Aldh1a7	0.9697	0.0008	Maff	0.9757	0.0010
20	Abcc3	0.9721	0.0008	Fabp4	0.9734	0.0009

**Table 9:** Genes ranked by the importance (based on the measure given by *R*), for Microarray and RNA-Seq, using the whole data including 8 verities of MOAs with the complete gene set

Rank	Microarray			RNASeq		
	Gene name	Resulted accuracy	*R*	Gene name	Resulted accuracy	*R*
1	LOC100912602	0.9616	0.0096	LOC690286	0.9407	0.0098
2	Il1rap	0.9821	0.008	Plcd3	0.9913	0.0087
3	Htatip2	0.9736	0.0074	Sgcb	0.9732	0.0078
4	Cd276	0.9557	0.0073	Retsat	0.9733	0.0077
5	Ankrd33b	0.9637	0.0065	Zfp39	0.9924	0.0076
6	Id1	0.9836	0.0064	Abcg5	0.9745	0.0074
7	Hgd	0.9649	0.0062	perja	0.9927	0.0073
8	RGD1305928	0.9562	0.0059	Sgk2	0.9530	0.0073
9	Acot2	0.9848	0.0052	Naaladl1	0.9657	0.0072
10	Dusp1	0.9860	0.0040	Mrps18b	0.9830	0.0071
11	Sat2	0.9870	0.0040	flergar	0.9842	0.0067
12	Adcy4	0.9663	0.0038	Nol3	0.9933	0.0067
13	Rexo4	0.9863	0.0037	stukaw	0.9755	0.0065
14	Dtnb	0.9863	0.0037	Igf2bp2	0.9837	0.0064
15	Hbb	0.9873	0.0037	slakoy	0.9937	0.0063
16	Fam111a	0.9676	0.0034	Serpinb1a	0.9852	0.0058
17	LOC690020	0.9770	0.0031	Ccnd1	0.9856	0.0054
18	Ddias	0.9870	0.0031	Id1	0.9947	0.0053
19	Resp18	0.9779	0.0031	Nrxn2	0.9947	0.0053
20	Mlc1	0.9879	0.0030	LOC494499	0.9658	0.0053

Referring to the Table 6, we observe that five of ten most important genes for classification (Cyp1a1, Fam111a, Ugt2b, Akr1b8, and Hbb) are in common between the two platforms, when the adjusted test set is used with the common set of gene. From literature search we found that Cyp1a1 encodes a member of the cytochrome P450 super-family of enzymes which catalyze many reactions involved in drug metabolism [7]. Likewise, Ugt2b belongs to a large family of proteins capable of detoxifying a wide variety of both endogenous and exogenous substrates such as biogenic amines, steroids, bile acids, phenolic compounds, and various other pharmacologically relevant compounds including numerous carcinogens, toxic environmental pollutants, and prescription drugs [8]. The function of Akr1b8 implicated in the pathogenesis of diabetic complications [9]. Mutations in Hbb have been implicated in a number of blood disorders [10], while mutations of Fam111a are strongly associated with type 2 Kenny-Caffey syndrome [11].

Table 7 presents the top 20 genes detected from complete gene sets for two platforms. We notice that 6 genes (Fam111a, Cyp1a1, Hbb, Aldh1a7, Psat1, and Obp3) for the microarray and 5 genes (Fam111a, Hbb, Cyp1a1, Ugt2b, and Dhrs7) for the RNASeq are in common with the top 20 of the previous analysis (Table 6).

Although the main goal of detecting impotent genes with the full data (Analysis 3.2) was to identify sets of genes making considerable impact on classifying all eight MOAs, interestingly, the outcome of this study (Tables 8 and 9) reveal high average (unpermuted) prediction accuracies (close to 100%) for both platforms using the 5 fold cross-validation technique. Tables 8 and 9 show lists of top genes ranked by the relative reduction of accuracy (*R*), for microarray and RNASeq, respectively. Clearly, there is no single gene that makes a substantial contribution to the accuracy. However, we identified two genes (Cyp1a1, Abcc3) that are commonly present in both lists when the complete set of genes was used. Based on the same analysis but performed using complete sets of genes we observe only one gene named Id1 is common important gene for the two platforms. We observed that Abcc3 is a member of the superfamily of ATP-binding cassette (ABC) transporters, which is involved in multi-drug resistance [12]. The Id1 gene plays a crucial role in activating hepatic stellate cells (HSCs) responding to liver damages [13].

## Methods

### Ensemble classifier

Support Vector Machines (SVM), Random Forests (RF), Neural Network (NN), Linear and Quadric Discriminant Analysis (LDA, QDA) are examples of standard techniques that are widely applied in classification problems. Performances of these classifiers are highly variable across problems. Thus, none of standard classifier can be considered to be the best for all classification settings. In complex situations, such as classifications in high dimensional genomic data, a more meaningful approach would be use an ensemble classifier which combines many standard classification algorithms together to develop an improved classifier. The ensemble classifier we use builds a number of individual models on randomly selected subsets of data which can then be combined or averaged in some meaningful fashion. Majority voting is a popular choice is for a typical solution. Such a classifier by allowing data based utilization of a multitude of classification algorithms for a upholds consistent performance in various types of data and classification problems. In this work, we use the adaptive optimal ensemble classer developed, via bagging and rank aggregation [14]. In this approach, several user specified classifiers are trained on bootstrap samples drawn from the original data using simple random sampling. Since the sampling is done with replacement, some samples will be repeated multiple times while others will be out of the bootstrap sample (known as out-of-bag (OOB) samples). Focusing on the prediction performances on the OOB samples, a best classifier is select based on various performance measures. For example, in a binary classification problem, sensitivity, specificity, and the area under the curve of the Receiver Operating Characteristic (ROC) curve are some legitimate performance measures. This method is equipped with rank aggregation [15, 16], which provides a great flexibility in selecting the optimal classifier with respect to various multiple performance measures. Predicted classes for a given test set is selected as the highest voted class, as predicted by the above set of “best" classifiers over all bootstrap resamples. Datta et al. [14], demonstrated the performance of the ensemble classifier using various numerical studies and real applications of gene expressions data. In the context of regression similar concepts have been developed [17].

The algorithm described below demonstrates the step by step procedure of developing an ensemble classifier [14]. Suppose the dataset of *n* samples with *p* dimensional covariates in the form of { ***X***_*n*×*p*_,***Y***_*n*×1_}, where ***X*** corresponds to independent variables and ***Y*** represents the dependent categorical variable that specifies a class label. Assume the ensemble classier is intend to built with *M* classification algorithms based on *K* different performance methods such as overall accuracy, class sensitivities etc. to optimize the predictive performance. Thus, we proceed as follows:

1. **Resampling:** Draw a bootstrap sample of size *n* $\left \{\boldsymbol {X}^{*}_{n \times p}, \boldsymbol {Y}^{*}_{n \times 1}\right \}$ from the original data {***X***_*n*×*p*_,***Y***_*n*×1_} by resampling rows with simple random sampling. Sampling is repeated until samples from all classes are present in the bootstrap sample and then determine the corresponding OOB sample that contains all samples which are left out from the bootstrap sample.
2. **Classifier Training:** Train *M* classification algorithms, *C*_1_,…,*C*_*M*_, on the bootstrap sample.
3. **Performance Assessment:** Obtain *M* predicted class labels for each OOB case. Since true classes of the OOB samples are known, calculate *K* different performance measures for each of *M* algorithms using their corresponding predictions.
4. **Rank Aggregation:** Rank *M* algorithms according to *K* performance measures. So, we have *K* ordered lists (*L*_1_,…,*L*_*K*_) of size *M*. These lists are then rank-aggregated using the weighted rank aggregation to determines the best algorithm *C*_(1)_ overall.
  Repeat the above procedure (steps 1–4) for *B* times, where *B* considered to be a large integer which is usually selected according to the computational capacity.
5. **Prediction for a New Sample:** Predict the class variable *Y* for a new sample *X* using the *B* prediction models $C^{1}_{(1)},\ldots,C^{B}_{(1)}$ and determined the highest voted class to obtain the final class prediction $\hat {Y}$.

### Rank aggregation

Suppose the performances of *M* classifiers are evaluated on the basis of *K* performance measures. Assume we have ordered lists *L*_1_,…,*L*_*K*_, where *i*th ordered list *L*_*i*_, *i*=1,…*K*, provides ranks of *M* algorithms on their performances evaluated on the *i*th measure. The rank aggregation [15, 16] procedure provides a single ranked list of *M* classifiers that minimizes the weighted sum of distances from all individual lists, given by the following objective function,

1 $$ \Phi(L)=\sum_{i}w_{i}d(L,L_{i})\text{,}   $$

where *L* is any possible ordered list of the *M* classifiers, *w*_*i*_’s are weights which represent the user specific importance of each of the *K* performance measures. The classifier in the first position of this aggregated list that is the optimal classifier overall with respect to all the validation measures. Of course, the default choice would be to use *w*_*i*_=1 for all *i* which means all the validation measures are taken as equally important in determining the optimal algorithm. Throughout out analyses, we have used *w*_*i*_=1. *d* is a distance function such as Spearman’s footrule or Kendall’s tau, which measures the closeness between two ordered lists. In this work, we use Spearman’s footrule distance function as the distance measure.

Often for high dimensional data, standard classifiers are combined with dimension reduction, variable selection, or penalization techniques such as Partial Least Squares (PLS), Principle Component Analysis (PCA), Random Forest (RF) based importance measures, *L*_1_ regularization, etc., for greater applicability and improved prediction accuracy [18, 19]. For a genomic data characterized by high dimension, use of an ensemble classifier developed on such set of improved component classifiers represents an ideal choice.

### Rat liver data

Our data for this study was released by 2015 CAMDA competition. Microarray and RNASeq platforms contain gene expression measurements of nearly 31,000 and 46,000 genes, respectively. The dataset consists of gene expression responses profiled by Affymetrix microarrays and Illumina RNASeq sequencer in rat liver tissues from 105 male Sprague-Dawley Rats, which are exposed to 27 different chemicals represented by 9 different MOAs. In the original experiment, a training set is formed with 45 rats, which are treated with 15 chemicals corresponding to MOAs of “PPARA", “CAR/PXR", “AhR", “Cytotoxic", “DNA damage", and 18 controls. Test set contains data on 36 rats which are treated with 12 chemicals corresponding to “PPARA", “CAR/PXR", “ER", “HMGCOA" and 6 controls. We found that two MOAs, “ER" and “HMGCOA" are present only in the test set. We further noticed that approximately 22,253 average expressions per sample in RNA-seq data were recorded as “NA", which indicates that insufficient number of reads mapped onto the gene to provide a reliable gene expression estimate. We retained gene sets of sizes 13,686 and 16,133 for microarray and RNASeq platforms, after (i) removing unnamed genes, (ii) removing genes with unobserved expressions, and (iii) averaging multiple expressions reported from the genes with unique names.

In this work, we used normalized expression levels that came from microarray data using Robust Multi-Array Average (RMA) expression measurements [20], whereas data obtained for RNASeq was already normalized via the Magic normalization [6, 21]. We decided that it would be reasonable to perform separate analysis with a common set of genes (8336) represented in both platforms and also with complete sets of genes, for a comparative study.

### Concordance experiments

We conducted three types of investigations for studying the performance of the proposed classifiers.

1. Train classifiers and make predictions on individual platforms.
2. Train classifiers in one platform to make predictions on the other platform.
3. Identify important variables (genes) for accurate classification.

In the 1st analysis, we explore the predictability of MOAs using various classifiers developed in the given training data. To our knowledge, there is no established criteria to define prediction for an unknown class that was not represented in the training data. Thus, we select an adjusted test set after eliminating all test samples belonging to two classes of “ER" and “HMGCOA", where the new test was used in parts of 1st and 3rd analysis. However we also considered the originally given test set as a part of 1st analysis by adopting following alternative classification approach. Accordingly, first we designated both “ER" and “HMGCOA" samples belonging to the original test set as “OTHER". For each classifier, then we determined the maximum class probability for a given test sample and if the above probability was less than 0.5 we selected the predicted class as “OTHER", else kept the originally predicted class. For this purpose, class probabilities for the ensemble classifier was calculated using the predicted class proportions observed in the *B* bootstrap samples.

Our objective with the 2nd analysis was to examine the inter-platform concordance between microarray and RNAseq platforms. Thus, we trained classifiers on a selected platform using the full dataset that included the both given training and test sets for making predictions on the other platform. However, since the classifier needed to run on both platforms for this analysis, each gene expression measurement was standardized, separately for both platforms, prior to the analysis.

For analyses 1 and 2, we selected an ensemble classifier developed with a set of *M*=7 standard classifiers, SVM, RF, LDA, PLS+RF, PLS+LDA, PCA+RF, PCA+LDA, and Recursive Partitioning (RPART). Primarily, classifiers are selected based on the prior information of their suitabilities in high dimensional data classification. Based on accuracies of predicted classes, each classifier was ranked for *K* number of performance measures (for example, overall accuracy, class specific accuracies ect.). Since the selection of performance measures for a multi-class classification problem is highly depend upon the aim of study; we optimized the overall prediction accuracy, and the class specific accuracy of each group for the 1st analysis. Furthermore we considered these performance measures to be equally important for classification (i.e., we used equal weights of *w*_*i*_=1, in Eq. (1)), whereas in the 2nd analysis in cross platforms, we focused only on the overall accuracy without optimizing multiple group specific performances. For these analyses, we chose *B* to be *B*=300. We performed a 10 fold cross-validation for each individual classifier to select the number of components for PLS and PCA methods, separately for two platforms. Assuming consistent performance in bootstrap samples similar to the original training data, we employed the same number of components to develop the ensemble classifier.

The 3rd analysis on identifying important variables is subdivided into following two parts.

1. Detecting important genes with the adjusted test set.
2. Detecting important genes with full data using the cross-validation method.

We applied a classifier on the perturbed training data resulted from randomly permuting gene expressions of a given gene to quantify its impact on the predictability of MOAs in a test set. Accordingly, each gene was ranked by a measure given by magnitude of accuracy reduction compared to the true accuracy (in unpermuted data), such that the rank 1 corresponds to the gene that has the highest negative impact on the overall prediction accuracy. In order to reduce the computational burden, we did not use the ensemble classifier for this purpose. Instead the component classifier PLS+LDA which had an overall accuracy close to that of the ensemble classifier was used. We performed theses analysis separately for both platforms to determine a common set of genes presented among the top 20 genes in both platforms.

For Analysis 3.1, we randomly permuted a gene’s expressions in the training set and then made predictions for the test set (adjusted test set) using the classifier trained on the permuted training data. The permutation procedure was repeated *l* times for each gene to calculate an average overall prediction accuracy (*A*). Finally, genes were ordered by *A*, ascending order. Here we chose *l* to be *l*=30 in order to achieve reasonably stable approximation, while keeping the computational costs in check.

Analysis 3.2 was performed using the full data which contained both originally given training and test sets. Here we applied the 5 fold cross-validation technique in order to evaluate the effect of each gene on classifying MOAs. Our approach consisted of two layers of randomization. For the *j*th, *j*=1,...,*J*, outer randomization, we randomly partitioned the dataset into 5 folds and selected a training set of 4 folds, while remaining fold was chosen as a test set. After randomly permuting the expressions of a given gene *i* across the above specified training set, a classifier was trained to predict on the selected test set. Now using the same approach we described in the previous part (Analysis 3.1) we obtained an average overall prediction accuracy $(A^{cv}_{i_{j}})$ by repeating the permutation *l* times. After that, the whole procedure was repeated *J* times for various random partition sets to obtain an average overall prediction accuracy ($A^{cv}_{i}$) for *i*th gene, based on all *J* scenarios.

$$A^{cv}_{i}=\frac{1}{J}\sum\limits_{j=1}^{J}A^{cv}_{i_{j}}. $$

Suppose *A* ^*cv*^ is the average true accuracy (unpermuted data) based on *J* random partition sets. Note that the magnitude of *A* ^*cv*^ can be varied. Thus a better measure will be a relative accuracy reduction (*R*_*i*_) given by,

$$R_{i}=\frac{A^{cv}-A^{cv}_{i}}{A^{cv}}, $$

where large values of *R*_*i*_ indicate high impacts on the classification. For Analysis 3.2, we used values *l*=30 and *J*=100, which stabilize the calculations without being computationally burdensome.

## Discussion

In this study, we used an ensemble classifier built on a set of standard classifiers to predict the MOA in Rat liver experiment data profiled by both microarrays and RNASeq. The newly constructed ensemble classifier performed reasonably well in both platforms individually. Using a selected test set and a set of genes (those present in both platforms) we observe comparable overall predictability of MOAs in the two platforms with 75% and 67% accuracies for microarray and RNAseq, respectively. Similarly, we observe well matched accuracies of 50% for both platforms for the full test sets based on an alternative approach. In an earlier classification approach [6] applied on the same data, reported average overall accuracies of 58% and 61% for microarray and RNAseq, suggesting a slightly better predictability in RNA-seq. However outcomes of these two studies are somewhat incomparable due to the differences in the training and test data sets used. For example, we considered controls as another class, whereas in their analysis, controls were not considered as a separate class. Interestingly, once we trained classifiers to make predictions on cross platforms, the ensemble classifier provided 100% accurate predictions for all 8 classes presented in the whole experiment. This result exhibits a perfect cross platform concordance for the purpose of classification. Also, our study clearly demonstrates a high agreement between the individual classifiers’ performances in two genomic platforms. Except for few scenarios, the ensemble classifier performed the best with respect to the overall accuracy and other class specific measures, in all experiments. We observe widely different classification performances among standard classifiers, which reflects the unreliability of restricting to a single classifier in case of high dimensional classification problems. On the other hand, this also demonstrates the utility of the adaptive ensemble classifier which is expected to perform as good or better than the individual classifiers with respect to multiple performance measures.

## Conclusion

In this study, we explored the inter-platform concordance between microarray and RNASeq in their ability to classify samples based on genomic information, using data profiled by a Rat Liver experiment. We used an ensemble classifier built on a set of seven standard classifiers to predict the MOA in Rat livers. The ensemble classifier performed reasonably well in both platforms individually, resulting respective 75% and 67% accuracies for microarray and RNAseq on a selected test set. When we trained classifiers to make predictions on cross platforms, the ensemble classifier provided remarkable 100% accurate predictions. This study demonstrates a high agreement between individual classifiers’ performances in two genomic platforms. Additionally, we identified a set of important genes those specifies MOAs, by focusing on their impact on the classification.

## Reviewers’ comments

### Reviewer’s report 1: Yiyi Liu (yiyi.liu@yale.edu), Yale University

In this manuscript, the authors investigated concordance between microarray and RNA-seq in classifying samples based on gene expression profiles. They tested the performances of eight classifiers, including one ensemble method, and obtained very interesting results. Overall the reviewer is positive about the work. There are several minor concerns that the authors need to address.

1. I suggest the authors add descriptions on the weights (*w*_*i*_’s) they used in rank aggregation of the ensemble classifier. The authors explained the main idea of the aggregation method, but explicitly stating all the parameters could improve the readability of the paper.
2. The authors mentioned RNA-seq data are “normalized via the Magic normalization”. I suggest citing the normalization method paper for reference. method.

Authors’ response:

1. *We have described the role and the choice of the weights.*
2. *The two suggested references have been added.*

### Reviewer’s report 2: Partha Dey (pdey.bit@gmail.com), Academy of Technology at Adisaptagram, Hooghly, India

The article “Inter-platform Concordance of Gene Expression Data for the Prediction of Chemical Mode of Action” by Siriwardhana et al. studies the consistency of the cross-platform classification accuracy between microarray and RNASeq in their ability to classify samples based on genomic information. Seven classifiers and an adaptive ensemble classifier developed around them were used to predict the Chemical Modes of Actions (MOA) on Rat Liver samples. The article is well written and nicely organized. In addition, addressing these few points should increase the impact of the research work across various spectrum of readers:

1. The “Results” section comes before the “Methods” section; if this is not due to some restriction of the publishers or typical of the field of investigation, the sequence may be reversed (to corroborate with usual practice in most research articles: after Methodology should come Results).
2. In the “Methods” section: The authors have mentioned the use of ’sampling with replacement.’ It would be relevant here to state the specific advantage of sampling with replacement as compared to sampling without replacement (which would result in a partition of the original training set into a pure-training and a in house-testing subsets– instead of some repeated data in the training samples and OOB samples for measuring the performance of the classifier). A brief description of the details of the different classifiers (viz. SVM, RF, LDA, PLS+RF, PLS+LDA, PCA+RF, PCA+LDA, and RPART), e.g. how many PCs were taken (or at least their range across different cases), whether linear or non-linear SVs, binary or n-ary partitioning, etc. may be provided to assist later users in this field to know the optimum classifier parameters.
3. In the “Discussion” section: Could a clue be given as to why the Ensemble classifier performed worse than at least one intrinsic classifier in those few scenarios? In particular, is a better ensemble approach possible, or is it in general impossible to have an ensemble classifier that performs best on all performance indices? It would be nice to have a commentary summarizing the important genes reported in Tables 6, 7, 8 and 9. For example those genes that appeared in most of the tables in Microarray or RNASeq, or both might be listed to enable the biologist to get the condensed information from this study.

Authors’ response: *Sampling with replacement is part of bootstrap which is standard procedure in bagging. An advantage is that training data of the same size as the original can be created and also the out of bag samples can be used as test data in a natural way. The classifiers were described in Datta et al. (2010). The tuning parameters were selected by cross validation as described in the paper. The ensemble classifier is optimal in an overall sense as defined by the rank aggregation procedure. It may not be possible to beat every individual classifier with respect to every performance measure. We have commented on some genes in Section “* *Importance of genes* *”.*

## Abbreviations

DEGs: Differentially expressed geans
LDA: Linear discriminant analysis
MOA: Chemical mode of action
OOB: Out of bag
PCA: Principle component analysis
PCA+LDA: Linear discriminant analysis with principle component analysis
PCA+RF: Random forest with principle component analysis
PLS: Partial least squares
PLS+LDA: Linear discriminant analysis with partial least squares
PLS+RF: Random forest with partial least squares
RF: Random forest
RPART: Recursive partitioning
SVM: Support vector machine
